# Age-related transversal changes in craniofacial sutures of the anterior viscerocranium in growing rats

**DOI:** 10.3389/fphys.2023.1201990

**Published:** 2023-06-16

**Authors:** Hande Gorucu-Coskuner, Mustafa Al-Yassary, Kelly Billiaert, Stavros Kiliaridis, Gregory S. Antonarakis

**Affiliations:** ^1^ Department of Orthodontics, Hacettepe University, Ankara, Türkiye; ^2^ Division of Orthodontics, University Clinics of Dental Medicine, University of Geneva, Geneva, Switzerland; ^3^ Department of Orthodontics, University of Bern, Bern, Switzerland

**Keywords:** micro-CT, rat, skull, suture growth, suture width, suture height

## Abstract

**Objective:** To evaluate the dimensional changes that occur in the internasal and nasopremaxillary sutures, and related transverse craniofacial dimensions, of rats from 4 to 38-weeks of age.

**Methods:** Four groups of twelve male Wistar rats were sacrificed at different ages [4-weeks (immature), 16-weeks (adolescent), 26-weeks (young adult), 38-weeks (adult)]. The rats were scanned with a high-resolution micro-computed tomography imaging device with 90 µm voxel size and 45 mm × 45 mm field of view (FOV) to obtain images of the viscreocranium, and with 10 µm voxel size and 5 mm × 5 mm FOV to obtain images of the internasal and left nasopremaxillary sutures. The nasal bone width, transverse width between the nasopremaxillary sutures and interzygomatic width were measured as craniofacial measurements. The endocranial, ectocranial and mean suture widths (cross-sectional area between endocranial and ectocranial borders/suture height), and suture height were measured at 5 frontal planes with 1.2 mm intervals. Outcomes were compared at different ages, and correlation coefficients were used to assess the relationship between craniofacial and suture changes.

**Results:** All transverse craniofacial dimensions increased significantly from 4–16 weeks of age (*p* < 0.001). After 16-weeks of age, the only significant increase was observed in interzygomatic width (*p* = 0.02), between 26 and 38 weeks. In both the internasal and nasopremaxillary sutures, the endocranial suture mean widths decreased from 4–16 weeks (*p* < 0.001 and *p* = 0.002, respectively), but did not show any significant change after 16-weeks of age. The ectocranial internasal suture width decreased from 4–16 weeks (*p* < 0.001), increased until 26-weeks (*p* = 0.035), and subsequently decreased (*p* < 0.001). The nasopremaxillary suture widths decreased from 4–38 weeks to varying degrees in different frontal planes. Except for the internasal ectocranial suture width, all suture measurements were found highly and negatively correlated with the transverse craniofacial dimensions. The height of the sutures increased with time, with the most significant changes occurring between 4 and 16 weeks of age (*p* < 0.001).

**Conclusion:** Although the internasal and nasopremaxillary endocranial suture widths nearly reach their final widths during adolescence, the changes in the ectocranial and mean suture widths continue into early adulthood. These results may serve as a reference for future studies aiming to evaluate the effects of functional demands on suture development and dimensional changes of the viscerocranium.

## Introduction

Craniofacial sutures are the fibrous articulations found between approximating osteogenic fronts of the craniofacial bones (Manlove et al., 2020). During postnatal growth, craniofacial sutures serve as the major growth sites for bone formation (Opperman, 2000). Throughout this period, new bone is added incrementally across the craniofacial sutures with remodelling, in order to establish the final cranial bony skeleton (Manlove et al., 2020). It is known that the sutures need to remain patent to function as bone growth sites (Opperman, 2000), while also undergoing a maturation process throughout life. In humans the cranial sutures close by fusion after the growth period (Wagemans et al., 1988). However, in the facial complex, the bones may remain separated by a fibrous union until the seventh or eighth decade of life (Opperman, 2000). In rats, all craniofacial sutures, except for the posterior interfrontal suture, remain patent throughout life (Bradley et al., 1996). Correspondingly, in mice, even in the adult period, craniofacial sutures are found to be highly vascularized and associated with osteogenesis and bone turnover (Luo et al., 2019).

The growth of facial sutures and the factors that affect their development is of major interest for orthodontists, as a common treatment choice for skeletal malocclusions in growing children includes dentofacial orthopaedic treatment with the aim of modifying sutural growth. Sutural growth is controlled by genetic and environmental factors, and the morphology and dimensions of individual sutures are related to the developmental adaptation occurring as a result of local mechanical stimulus (Vij and Mao, 2006; Savoldi et al., 2019). Accordingly, even suture shape (interdigitating or abutting) has been found to be related to the associated masticatory strain (Rafferty and Herring, 1999). In the rat model, the nasopremaxillary and internasal sutures are commonly-evaluated sutures and are clinically important as they are potentially affected by the forces exerted by the masticatory muscles (Engstrom et al., 1986; Katsaros et al., 1994; Katsaros et al., 2006), and the growth changes in these sutures resembles transverse growth of the facial bones. Although transverse growth deficiency of the maxilla is one of the most common skeletal abnormalities in the craniofacial region and can lead to malocclusions, the transverse dimension remains the least understood from the three dimensions of craniofacial growth (Yi et al., 2021).

Several studies have been undertaken in an attempt to understand the effects of different functional demands on sutural growth of the anterior facial area (Engstrom et al., 1986; Kiliaridis, 1986; Katsaros et al., 1994; Kiliaridis et al., 1996; Katsaros et al., 2006). These studies mostly evaluate the differences in sutural growth changes under different functional demands. However, knowledge about the changes in sutural dimensions from early childhood to adulthood in rats under normal circumstances is lacking. Interpreting the natural dimensional changes of the craniofacial sutures would help us better understand the mechanisms underlying sutural growth, and evaluate the studies considering the effects of functional variations on growth from a different angle.

Facial sutures are patent at birth and gradually ossify at variable rates (Wang et al., 2022). Bone appositions in the osteogenic fronts of the sutures leads to an increase in facial dimensions. The maturation of facial sutures differs from the other sutures and they mostly remain patent through late adulthood (Martinez-Maza et al., 2013). Although facial sutures remain patent for a considerable duration, bony growth does not continue indefinitely (Wang et al., 2022). Apart from the factors that affect normal growth, fusion of the facial sutures or decreased rate of bone apposition at the osteogenic fronts of the sutures might lead to growth deficiency in related dimensions. In the light of the foregoing knowledge, the aim of the present study was to evaluate the dimensional changes that occur in the internasal and nasopremaxillary sutures and related craniofacial dimensions of rats from 4 to 38 weeks of age. Additionally, the relationship between the suture width and transverse dimensions of the upper viscerocranium was evaluated. In the present study, the growth changes of internasal and nasopremaxillary sutures were evaluated for a broader knowledge of the transverse growth of craniofacial structures of the viscerocranium.

## Materials and methods

The sample of the present cross-sectional study consisted of 48 male Wistar rats that were used for a previous study (approved by the local ethics committee for animal research: GE/15/20A & GE31) and sacrificed at different ages. The study groups were established as: 4-week-old rats (immature group; *n* = 12), 16-week-old rats (adolescent group; *n* = 12), 26-week-old rats (young adult group; *n* = 12) and 38-week-old rats (adult group; *n* = 12). All of the rats were housed two per cage in the animal facility, with the provision of standard rat chow in the form of pellets and water *ad libitum*. Conditions in the animal facility were a 12-h light/dark cycle. At the ages of 4, 16, and 26 weeks, and 38 weeks, the animals to be sacrificed were anesthetised by inhalation of isoflurane (5%), and subsequently an intraperitoneal injection of pentobarbitol (150 mg/kg diluted to 200 mg/mL) was administered. After the rats were sacrificed, the heads were resected from the body and kept in 4% paraformaldehyde solution.

For the experiment, the heads were scanned with a high-resolution micro-computed tomography (μCT) device (Quantum GX micro-CT Imaging System) with two different scanning parameters. The first types of scans were taken to obtain the image of the viscerocranium, with 90 µm voxel size and 45 × 45 mm field of view for 14 min (x-ray tube potential: 90 kV, current: 40 µA). The following measurements were performed with the OsiriX MD software (Rosset et al., 2004), by using the DICOM files obtained from these scans.

**FIGURE 1:**
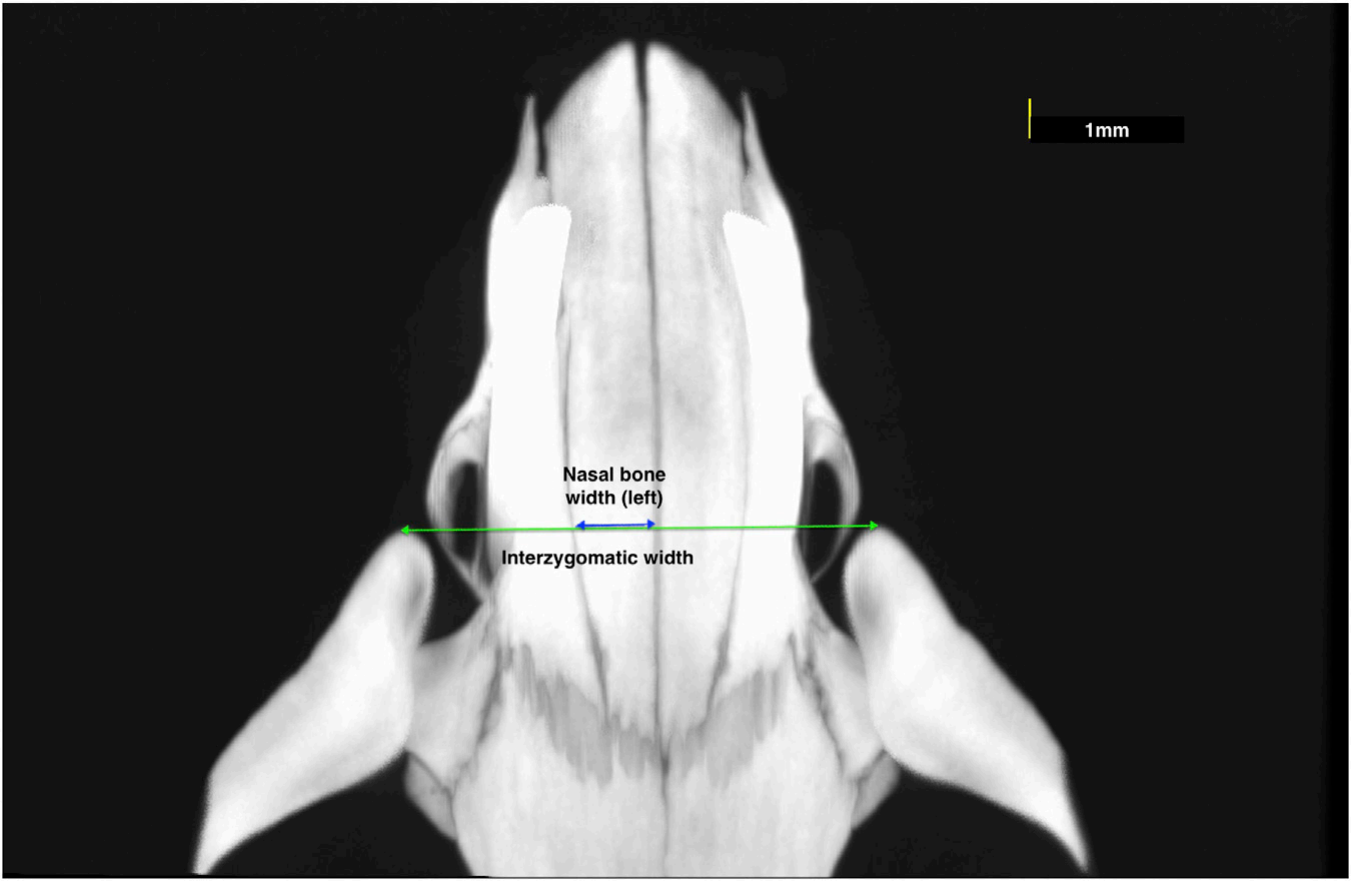
Measurements representing the nasal bone width and interzygomatic width.

**FIGURE 2:**
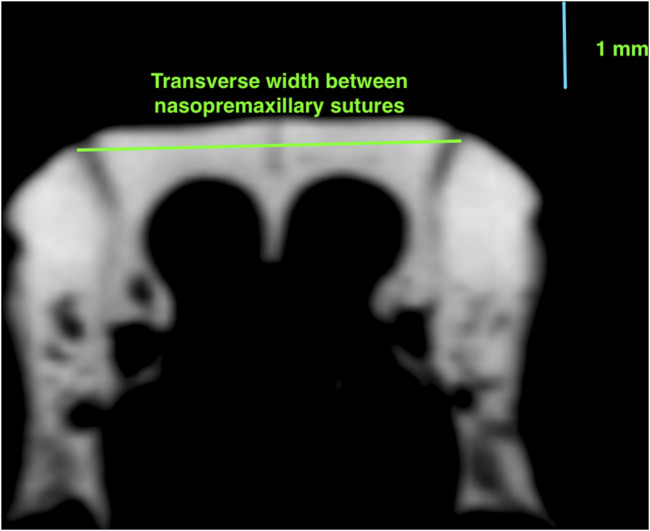
Measurements representing the transverse width between nasopremaxillary sutures.

1. Nasal bone width: the mean width of right and left nasal bones at the level of the most anterior point of zygomatic arches (Figure 1);
2. Interzygomatic width: the transverse width between the most anterior points of the right and left zygomatic arches (Figure 1);
3. Transverse width between nasopremaxillary sutures: the width of premaxilla between the nasopremaxillary sutures at the level of the most anterior point of the zygomatic processes (Figure 2).

**FIGURE 3:**
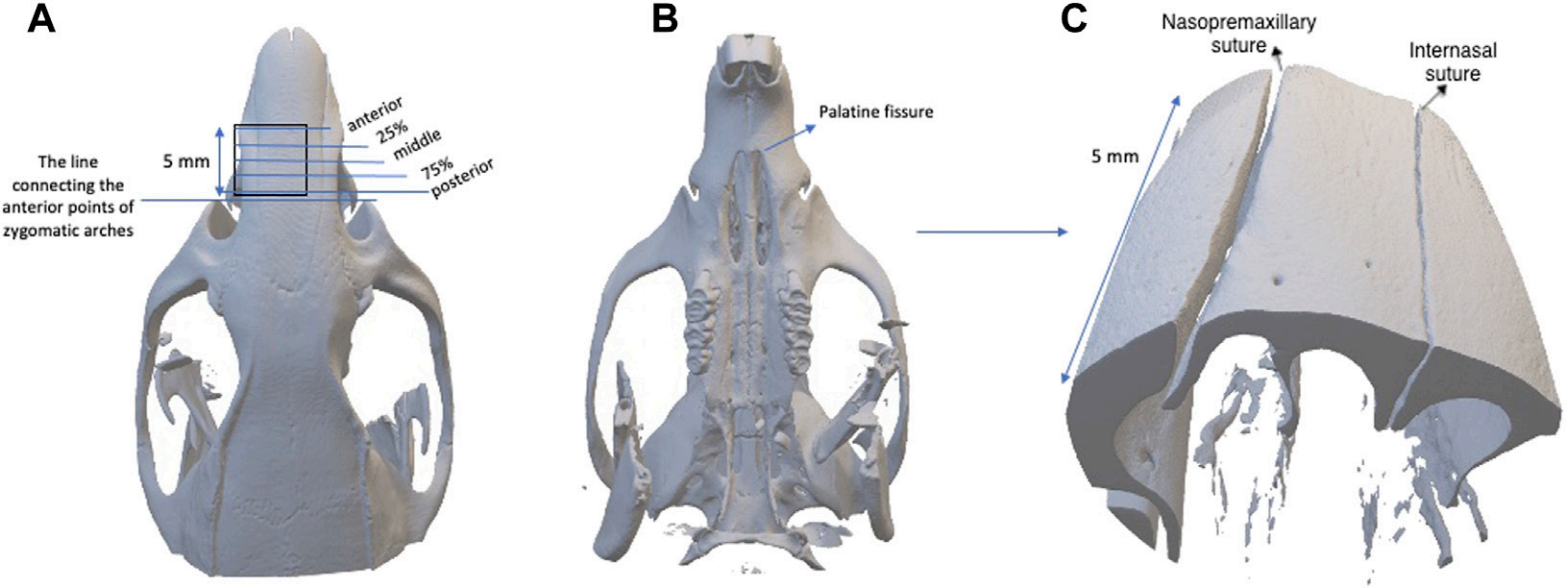
**(A)** The region of interest starting from the most anterior point of zygomatic arches and extending 5 mm anteriorly, and the placement of 5 frontal planes used for the measurements of anterior, 25%, middle, 75% and posterior regions; **(B)** the caudal view of the skull; **(C)** the three-dimensional image established with the scanning protocol.

The second types of scans were taken to obtain images of the internasal and nasopremaxillary sutures, by using 10 µm voxel size and 5 × 5 mm field of view for 57 min (x-ray tube potential: 90 kV, current: 88 µA). Air was used as the scanning medium in both types of scans, and scanning was carried out without a filter. To obtain a standardized area for scanning, a pilot study was conducted to evaluate the dimensional growth changes of the nasal bone. For this purpose, the heads of twelve 4-week-old, six 16-week-old and nine 38-week-old rats were used for comprehensive nasal bone measurements, and it was seen that the distance between the zygomatic arches and the anterior point of the palatine fissure showed negligible change with increasing age. Therefore, the level of the zygomatic arches was identified and selected as the most posterior limit of reference in the scans. Accordingly, scans were performed using a standardized protocol, with the region of interest starting from the most anterior point of zygomatic arches and extending 5 mm anteriorly, in the axial plane, including the internasal and left nasopremaxillary sutures (Figure 3). In the sagittal plane the nasal bone was set parallel to the tube, and a foam mould was used to achieve this position during the scans. The following measurements were performed with the OsiriX MD software (Rosset et al., 2004), by using the DICOM files obtained from the scans.

**FIGURE 4:**
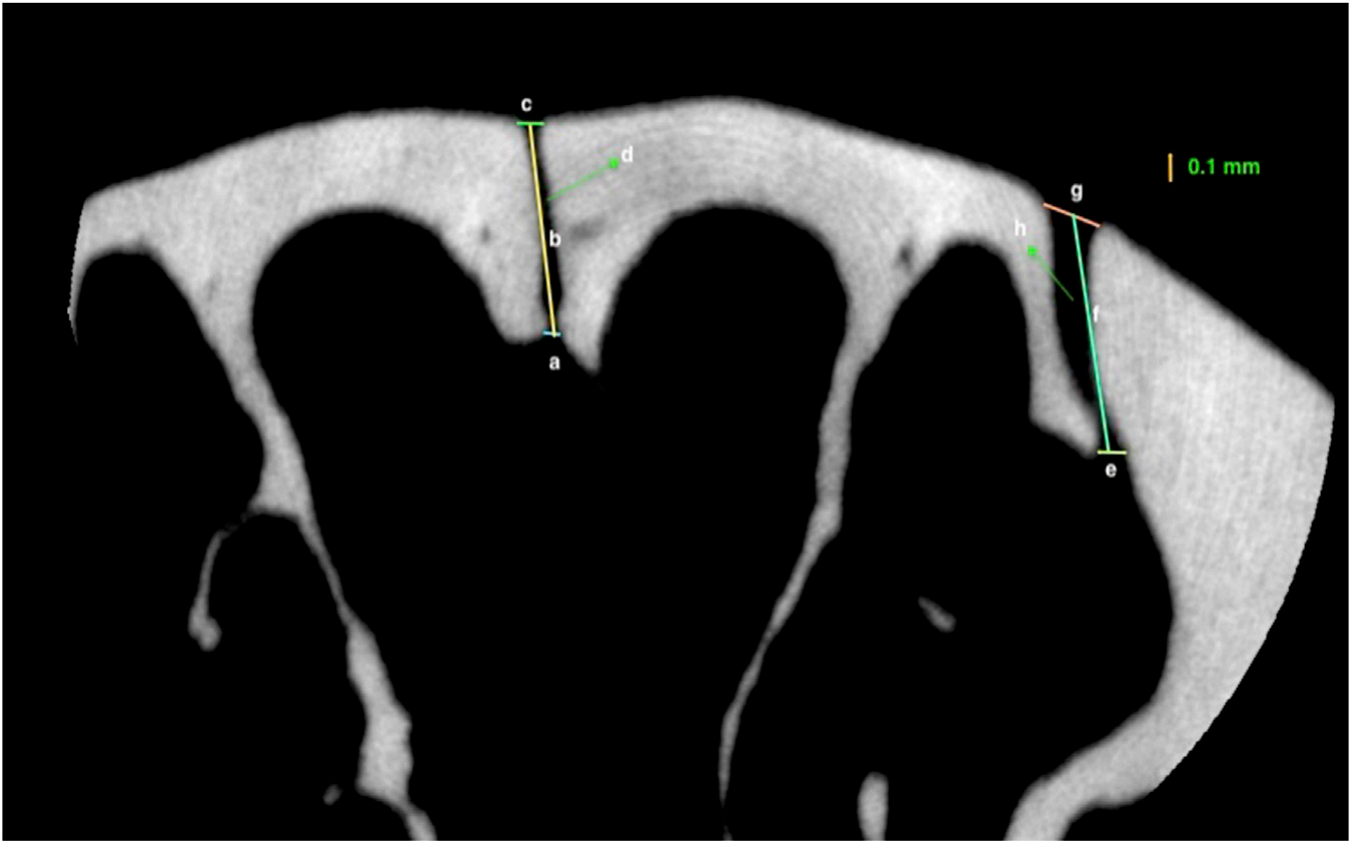
Measurements representing: **(A)** Endocranial suture width of the internasal suture; **(B)** Height of the internasal suture; **(C)** Ectocranial suture width of the internasal suture **(D)** The area of internasal suture; **(E)** Endocranial suture width of nasopremaxillary suture; **(F)** Height of the nasopremaxillary suture; **(G)** Ectocranial suture width of the nasopremaxillary suture; **(H)**; area of nasopremaxillary suture of a 26-week-old rat.

1. Endocranial suture width: the closest distance between the two most convex points of the endocranial bony borders (Figure 4);
2. Ectocranial suture width: the closest distance between the two most convex points of the ectocranial bony borders (Figure 4);
3. Suture height: the height between the middle points of endocranial and ectocranial suture lines (Figure 4);
4. Mean suture width: the intersutural area between the endocranial and ectocranial suture lines (Figure 4) divided by suture height (Figure 4).

The measurements were performed on 5 frontal planes with 1.2 mm intervals by one observer (H.G.C). The measurements were classified as posterior (0.1 mm anterior to zygomatic arch level), 75% (1.3 mm anterior to zygomatic arch level), middle (middle point of the scan), 25% (3.7 mm anterior to zygomatic arch level) and anterior (4.9 mm anterior to zygomatic arch level) as shown in Figure 3. During the statistical analysis, the average values of all measurements performed on 5 frontal planes were calculated for the total sutural evaluation, and all regions were analysed separately for the evaluation of particular areas.

### Statistical analysis

Data were analysed using SPSS Statistics Version 27.0 (SPSS, IBM, Armonk, New York). Means, standard deviations, and medians (including minimum and maximum values) were calculated for descriptive statistics. All data were tested for normality using the Shapiro-Wilk test, and for homogeneity of variance by Levene’s test. For comparisons between different ages, one-way ANOVA with Bonferroni correction was used for parametric variables, and Kruskal Wallis with Dunn’s correction was used for non-parametric variables. Pearson’s correlation coefficients were used to assess the relationship between craniofacial dimensions and suture dimensional changes at the level of the most anterior point of zygomatic arches. A threshold of *p* < 0.05 was considered statistically significant.

For an evaluation of the error of the method, the whole measurement procedure was repeated for 16 rats 2 weeks after the initial measurements by the same observer (H.G.C.). Intraobserver agreement was evaluated using the intraclass correlation coefficients (ICC) with 95% confidence intervals (CI).

## Results

Error of the method measurements showed ICC values ranging from 0.937 (95%CI = 0.781; 0.980) to 0.999 (95%CI = 0.996; 1.000) for the internasal suture and from 0.916 (95%CI = 0.758; 0.971) to 0.999 (95%CI = 0.996; 1.000) for the nasopremaxillary suture, indicating excellent intra-examiner reliability.

### Craniofacial dimensions

When the craniofacial dimensions were evaluated, it was seen that the nasal bone width significantly increased between 4 and 16 weeks of age (*p* < 0.001) (Table 1). Similarly, the interzygomatic width and the transverse width between the nasopremaxillary sutures increased significantly from 4 to 16 weeks of age (*p* < 0.001). After 16 weeks of age, the only significant increase occurring was for the interzygomatic width (*p* = 0.02), observed between 26 and 38 weeks of age.

**TABLE 1:** Transversal dimensions of the upper viscerocranium in rats of different ages.

	4-week-old (*n* = 12)	16-week-old (*n* = 12)	26-week-old (*n* = 12)	38-week-old (*n* = 12)	Intergroup comparison *p*-value	4–16 weeks	16–26 weeks	26–38 weeks
Nasal bone width (mean of right and left) (mm)	1.6 ± 0.1	1.9 ± 0.1	2.0 ± 0.1	2.0 ± 0.1	<0.001***	<0.001***	NS	NS
Transverse width between nasopremaxillary sutures (mm)	3.6 ± 0.16	4.45 ± 0.2	4.54 ± 0.21	4.55 ± 0.17	<0.001***	<0.001***	NS	NS
Interzygomatic width (mm)	8.7 ± 0.3	11.4 ± 0.5	11.6 ± 0.5	12.2 ± 0.4	<0.001***	<0.001***	NS	0.02*

One-way ANOVA, with Bonferroni correction was used (means ± standard deviation values are given).

*p < 0.05; **p < 0.01; ***p < 0.001; NS, not significant.

### Internasal suture

**FIGURE 5:**
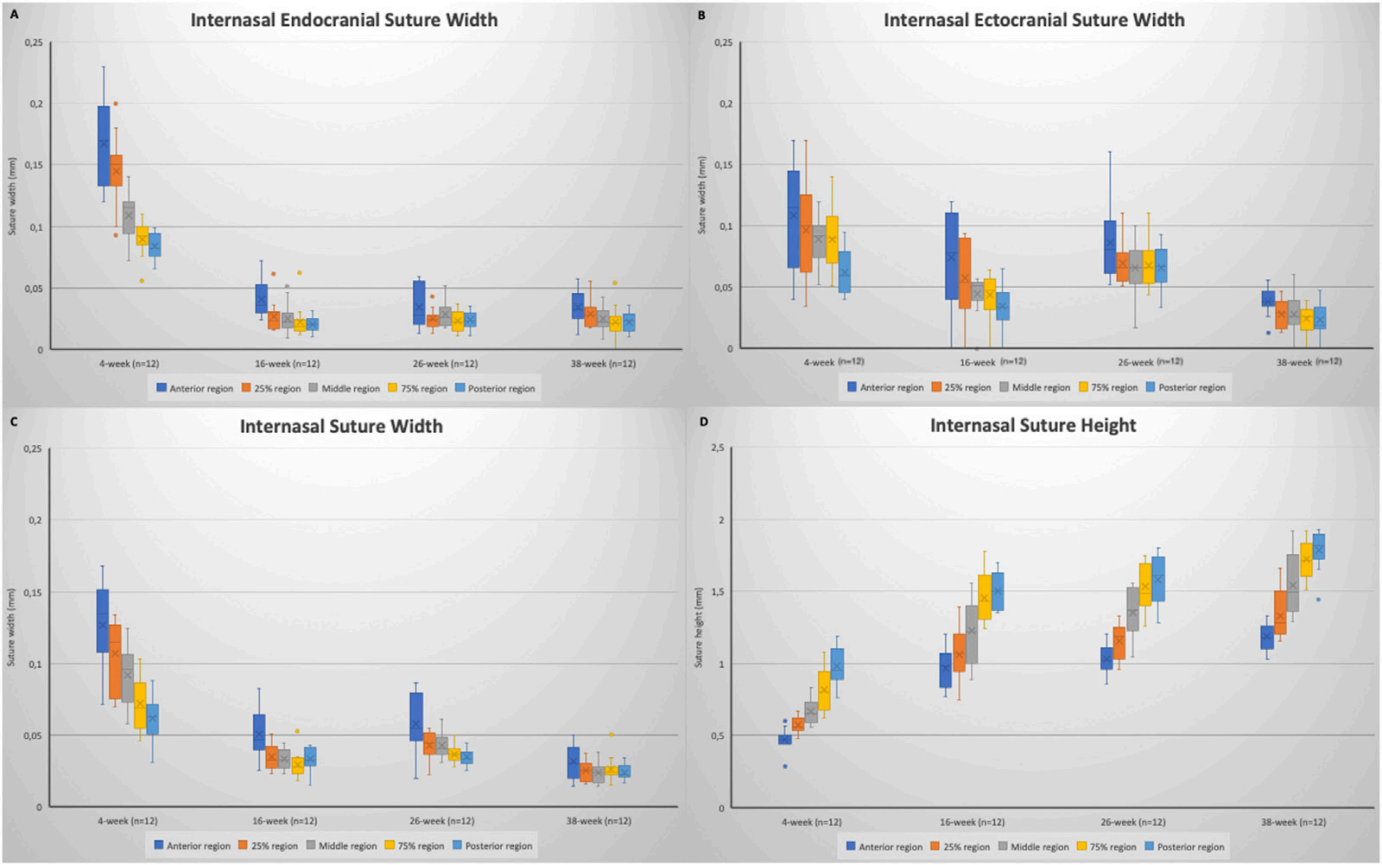
Box plots showing the changes in: **(A)** internasal endocranial suture width; **(B)** internasal ectocranial suture width; **(C)** internasal suture width (mean); **(D)** internasal suture height. Boxes represent the interquartile range, with whiskers representing the maximum and minimum values. The median is represented as the line within the box and the mean as the X within the box. Outliers are represented with individual data points.

**FIGURE 6:**
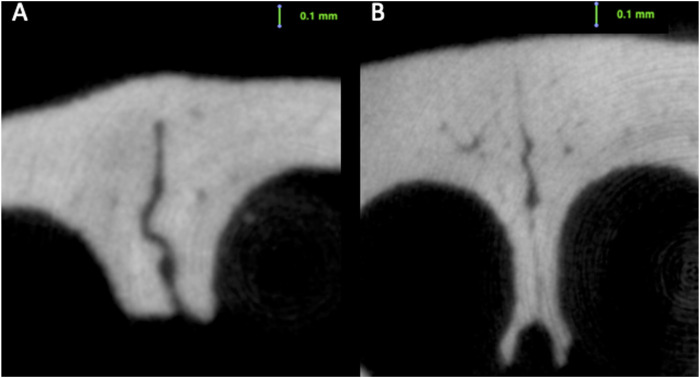
Fusion seen in the ectocranial region of the internasal suture of a **(A)** 16-week-old rat; and **(B)** 38-week-old rat.

When the internasal suture morphology was evaluated, although there were some small variations, suture width tended to decrease and suture height tended to increase moving from the anterior to the posterior region. With regard to changes over time, between 4 and 16 weeks of age, there was a general decrease in internasal suture widths (Table 2; Figure 5). More particularly, it was seen that endocranial (*p* < 0.001), ectocranial (*p* < 0.001) and mean suture widths (*p* < 0.001) significantly decreased in all regions except the anterior ectocranial region (Figure 5). Interestingly, 2 of the 16-week-old rats showed fusion in the 25% and middle regions of the internasal suture (Figure 6).

**TABLE 2:** Endocranial, ectocranial and mean widths, and height of the internasal sutures (mean values of all regions evaluated).

Internasal suture (mm)	4-week-old (*n* = 12)	16-week-old (*n* = 12)	26-week-old (*n* = 12)	38-week-old (*n* = 12)	Intergroup comparison *p*-value	4–16 weeks	16–26 weeks	26–38 weeks
Mean endocranial width	0.121 (0.092, 0.140)	0.025 (0.020, 0.043)	0.026 (0.016, 0.036)	0.026 (0.016, 0.043)	<0.001***^a^	<0.001***	NS	NS
Mean ectocranial width	0.089 ± 0.022	0.051 ± 0.017	0.071 ± 0.018	0.028 ± 0.007	<0.001***^b^	<0.001***	0.035*	<0.001***
Mean width	0.092 ± 0.020	0.036 ± 0.007	0.043 ± 0.007	0.026 ± 0.006	<0.001***^b^	<0.001***	NS	0.006**
Mean height	0.0704 ± 0.099	1.242 ± 0.155	1.332 ± 0.121	1.513 ± 0.129	<0.001***^b^	<0.001***	NS	0.007**

^a^ Kruskal Wallis with Dunn’s correction was used, with median (minimum, maximum) values given.

^b^ One-way ANOVA, with Bonferroni correction was used (means ± standard deviation values are given).

*p < 0.05; **p < 0.01; ***p < 0.001; NS, not significant.

Regarding the endocranial internasal suture widths, the only significant change was seen between 4 and 16 weeks of age, as a decrease (*p* < 0.001). After 16 weeks of age, the endocranial internasal suture widths did not show any significant changes with time. When ectocranial suture widths were evaluated, it was seen that after the significant decrease from 4 to 16 weeks of age, a significant increase was observed (*p* = 0.035), especially in the middle to posterior areas (Table 2; Figure 5). After 26 weeks of age, there was a tendency for the ectocranial suture widths to decrease again (*p* < 0.001). At 38 weeks of age, fusion was seen in the ectocranial area of the middle to the posterior regions and the endocranial area (close to the posterior region, 75%) of the internasal suture in one rat.

When the mean internasal suture widths were evaluated, a significant decrease was seen from 4 to 16 weeks of age (*p* < 0.001), reflecting both the decreases in endocranial and ectocranial suture widths. Following a relatively stable period for mean internasal suture widths between 16 and 26 weeks of age, a significant change, probably due to the decrease in ectocranial suture width, was observed from 26 to 38 weeks of age (*p* = 0.006) (Table 2).

Regarding suture height, a significant increase (*p* < 0.01) was observed from 4 to 16 weeks of age (Table 2; Figure 5). Although there was not a significant change in suture height from 16 to 26 weeks, the increasing pattern of suture height continued between 26 and 38 weeks of age (*p* = 0.007), especially in the anterior (*p* = 0.003), 75% (*p* = 0.032) and the posterior (*p* = 0.008) regions (Figure 5).

### Nasopremaxillary suture

**FIGURE 7:**
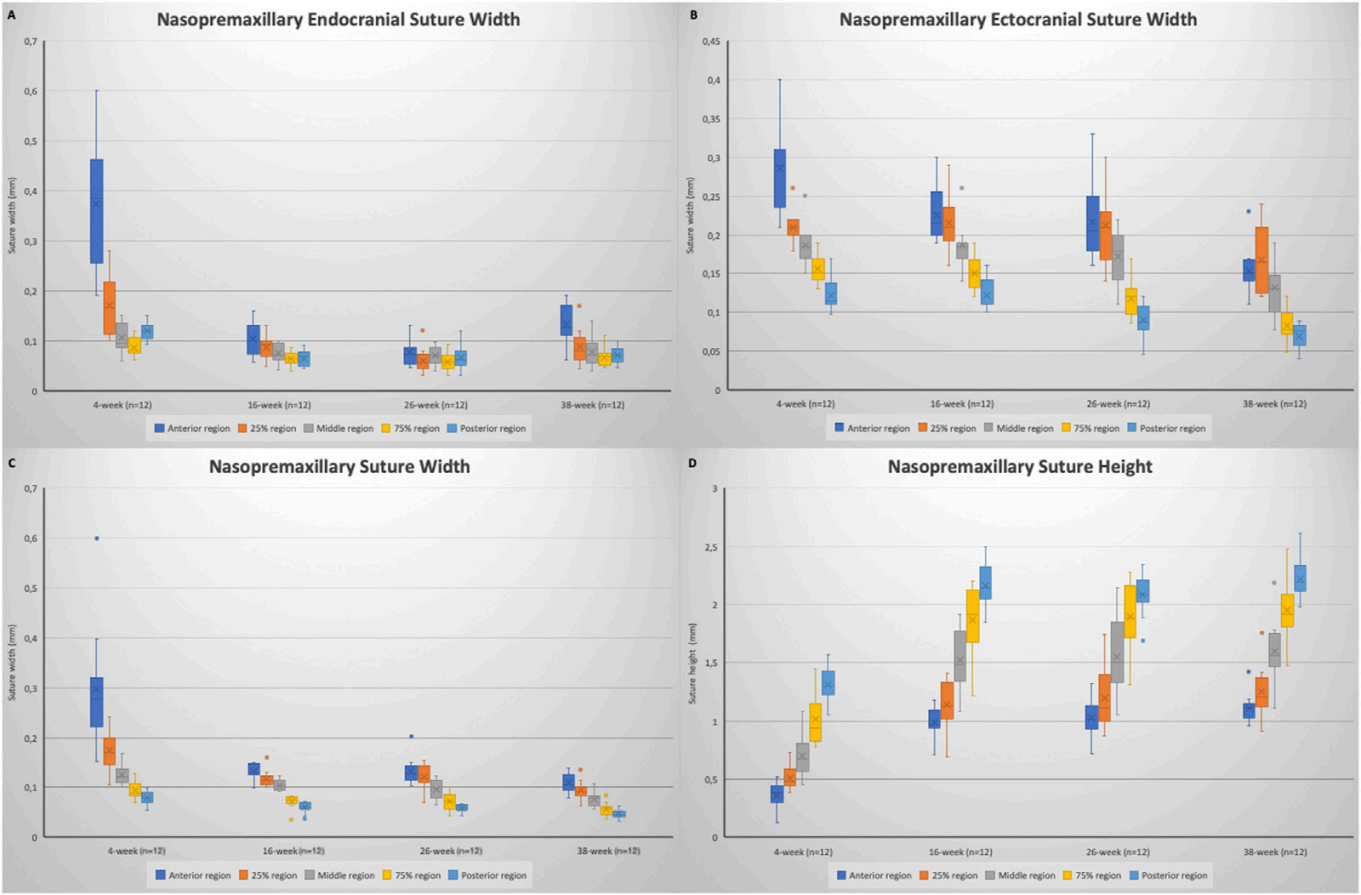
Box plots showing the changes in: **(A)** nasopremaxillary endocranial suture width; **(B)** nasopremaxillary ectocranial suture width; **(C)** nasopremaxillary suture width (mean); **(D)** nasopremaxillary suture height. Boxes represent the interquartile range, with whiskers representing the maximum and minimum values. The median is represented as the line within the box and the mean as the X within the box. Outliers are represented with individual data points.

When the nasopremaxillary suture morphology was evaluated, it was seen that the suture width tends to decrease and suture height tends to increase going from the anterior to the posterior region, excluding for the posterior endocranial area that showed bigger variation between the animals. When suture width changes were evaluated at different ages, it was seen that the widths significantly decreased from 4 to 38 weeks of age (Figure 7). This decrease however did not occur in a linear manner. The endocranial width of the nasopremaxillary suture significantly decreased from 4 to 16 weeks of age (*p* = 0.002), but no significant change was observed after 16 weeks of age (Table 3; Figure 7).

**TABLE 3:** Endocranial, ectocranial and mean widths, and height of the nasopremaxillary sutures (mean values of all regions evaluated).

Nasopremaxillary suture (mm)	4-week-old (*n* = 12)	16-week-old (*n* = 12)	26-week-old (*n* = 12)	38-week-old (*n* = 12)	Intergroup comparison *p*-value	4–16 weeks	16–26 weeks	26–38 weeks
Mean endocranial width	0.164 (0.051, 0.098)	0.079 (0.051, 0.098)	0.061 (0.051, 0.107)	0.081 (0.066, 0.134)	<0.001***^b^	0.002**	NS	NS
Mean ectocranial width	0.192 ± 0.021	0.180 ± 0.015	0.162 ± 0.029	0.121 ± 0.022	<0.001***^a^	NS	NS	<0.001***
Mean width	0.154 ± 0.037	0.097 ± 0.006	0.096 ± 0.016	0.076 ± 0.012	<0.001***^a^	<0.001***	NS	NS
Mean height	0.781 ± 0.134	1.534 ± 0.204	1.551 ± 0.214	1.630 ± 0.192	<0.001***^a^	<0.001***	NS	NS

^a^ One-way ANOVA, with Bonferroni correction was used (means ± standard deviation values are given).

^b^ Kruskal Wallis with Dunn’s correction was used, with median (minimum, maximum) values given.

*p < 0.05; **p < 0.01; ***p < 0.001; NS, not significant.

Regarding the ectocranial suture width, the decrease in width showed differences between different regions (Figure 6). Globally, maximum dimensional change of the ectocranial nasopremaxillary suture width started primarily from the anterior region, and then continued in the posterior region, followed by the middle areas. More specifically, the most significant decrease in the anterior ectocranial width was observed from 4 to 16 weeks (*p* = 0.009) and from 26 to 38 weeks of age (*p* = 0.005). Apart from the anterior region, no significant change was observed between 4 and 16 weeks of age. From 16 to 26 weeks of age, a significant decrease was observed in the posterior ectocranial areas of the nasopremaxillary suture, and between 26 and 38 weeks of age all ectocranial surfaces excluding the posterior region showed a significant decrease (*p* < 0.05), which also resulted in a decrease in mean ectocranial suture width (*p* < 0.001) (Table 3). Consequently, although there were some differences between regions, the dimensional changes of the ectocranial region continued from 4 to 38 weeks of age in the nasopremaxillary suture with the most significant changes occurring between 26 and 38 weeks of age.

When the mean suture widths were evaluated, the most significant decrease was seen from 4 to 16 weeks of age in all regions (*p* < 0.05), and between 26 and 38 weeks of age in the middle to posterior regions (*p* < 0.05) (Figure 6). Finally, the nasopremaxillary suture height showed a significant increase from 4 to 16 weeks of age (*p* < 0.001), and mostly remained stable after 16 weeks of age (Table 3; Figure 7).

### Correlation between the craniofacial dimensions and suture measurements

Correlation coefficients (r) between craniofacial dimensions and suture measurements, and their significance level, are presented in Table 4. Internasal endocranial suture width, mean suture width, and all nasopremaxillary suture width measurements were strongly and negatively correlated with all craniofacial dimensions (*p* < 0.01). The internasal ectocranial width was also strongly and negatively correlated with the interzygomatic width (*r* = −.421, *p* = 0.003). Apart from this strong correlation, the internasal ectocranial width was found to be negatively correlated with the nasal bone width (*r* = −.330, *p* = 0.022).

**TABLE 4:** Correlations between craniofacial dimensions and suture measurements.

	Internasal suture width	Internasal endocranial width	Internasal ectocranial width	Nasopremaxillary suture width	Nasopremaxillary endocranial suture width	Nasopremaxillary ectocranial suture width
Nasal bone width (mean of right and left)	−0.699 (<0.001***)	−0.830 (<0.001***)	−0.330 (0.022^a^)	−0.629 (<0.001***)	−0.658 (<0.001***)	−0.452 (0.001**)
Transverse width between nasopremaxillary sutures	−0.670 (<0.001***)	−0.868 (<0.001***)	−0.281 (0.053)	−0.571 (<0.001***)	−0.688 (<0.001***)	−0.392 (0.006**)
Interzygomatic width	−0.798 (<0.001***)	−0.889 (<0.001***)	−0.421 (0.003**)	−0.693 (<0.001***)	−0.714 (<0.001***)	−0.511 (<0.001***)

Values are presented as r(p) value.

Pearson correlation coefficient analysis was used, the significance level was *p* < 0.05.

*p < 0.05; **p < 0.01; ***p < 0.001.

## Discussion

Cranial suture biology can be readily studied in a rat model, as these animals are small, and the biologic behaviour of their craniofacial bones and sutures are well-described (VandeBerg et al., 2004; Vij and Mao, 2006; Abed et al., 2007). In the present study, male Wistar rats were used to evaluate the growth changes of internasal and nasopremaxillary sutures, from 4 to 38 weeks of age, representing immaturity to adulthood. Male rats were selected for the present study to overcome the concerns about confounding contributions from the oestrous cycle of female animals (Clayton and Collins, 2014), and to improve the homogeneity of the study. Rats have an accelerated childhood, compared to humans, as they are weaned at approximately 3 weeks of age, and become sexually mature at around 6 weeks of age (Sengupta, 2013). Based on this, the first measurements of this study, at 4 weeks of age, represent childhood at a prepubertal stage. Following adolescence (16 weeks of age), skeletal growth tapers off in male rats at approximately 7–8 months of age (Baker and Weisbroth, 1979), and thus the changes between 26 and 38 weeks of age in this study correspond to changes during young adulthood. Accordingly, in this study, it was aimed to describe the dimensional changes in the internasal and nasopremaxillary sutures in growing rats from childhood to adulthood.

In the present study, μCT imaging was used for the dimensional measurements of sutures. This helps the visualization of three-dimensional structures and is widely used to evaluate bone quality and morphology of small animals (Shim et al., 2022). High resolution μCT imaging can be used to evaluate small areas with high precision, and is found to be a useful tool for a greater understanding of the cranial suture as it is a non-destructive imaging process and structure-oriented slices can be produced easily for quantitative analysis of the sutures (Corega et al., 2010). Another advantage of this imaging method is overcoming the problems related to the difficulty in obtaining histological sections perpendicular to the suture (Rafferty and Herring, 1999). Several μCT studies (Gosain et al., 2011; Maloul et al., 2013; Savoldi et al., 2019; Koca et al., 2021; Nuri et al., 2022) have been conducted to evaluate suture widths during craniofacial growth, but previous studies on the dimensional changes occurring with growth in the internasal and nasopremaxillary sutures are lacking.

During μCT scanning for suture evaluation, the area of interest was chosen as the region starting from the most anterior point of the zygomatic arches extending 5 mm anteriorly. This area corresponds roughly to the middle of the nasal bone that was thought to be less influenced by bone appositions, as the length increase of the nasal bone was shown to be due to bone formation at the nasofrontal suture and the tip of the nose during growth of a rat (Vilmann, 1976). The chosen landmark was clearly visible when orienting the samples for the μCT imaging scans at all growth stages, making placement of the sample reproducible. Moreover, based on our pilot study, the distance between the zygomatic arches and the anterior point of the palatine fissure showed negligible change over time, which aids in the repeatability of the measurements. Correspondingly, the transverse craniofacial measurements were conducted at the level of zygomatic arches.

The transverse growth of the craniofacial region has a big importance in normal craniofacial growth, as maxillary transversal deficiency is the most common skeletal change that involves the maxilla, and can lead to occlusal disharmony and functional problems (Fastuca et al., 2015; Bin Dakhil and Bin Salamah, 2021). Several studies have therefore been conducted to understand the factors that can affect transverse growth of the craniofacial region (Katsaros, 2001; Katsaros et al., 2002; Odman et al., 2019). Bone apposition in the internasal and nasopremaxillary sutures contributes to the transverse growth of the facial structures. In the present study, growth changes of the transverse craniofacial dimensions and internasal and nasopremaxillary sutures were evaluated in an attempt to better understand this transverse growth pattern of growing rats from childhood to adulthood under normal conditions.

When the craniofacial dimensions were evaluated, it was seen that the width of the upper viscerocranium increased significantly from 4 to 16 weeks of age. After that period, a significant increase was observed only in the interzygomatic width. This increase in width can be enhanced from bone apposition because of the muscle attachments. However, when the changes in the transversal dimensions are evaluated, it can be hypothesized that the dimensional increase resulted from sutural growth mostly occurrs before 16 weeks of age.

Suture growth includes several parallel events such as an increase in suture width, sutural cell density and sutural osteogenesis (Kopher and Mao, 2003; Vij and Mao, 2006). Consequently, intramembranous bone is added to the edges of the bone fronts (Opperman, 2000), and the dimensions of the sutures change. The decrease of suture width has been assumed to be related to the decreased rate of bone apposition at the osteogenic fronts in previous studies (Katsaros et al., 2006), thus by extrapolation the dimensional changes of the suture might give us information regarding the rate of sutural growth. Changes in the width and growth rate of the sutures are of interest to orthodontists, as the success of orthopaedic treatments depend, to a large extent, on sutural growth and remodelling. In addition, to understand the factors that can lead to changes in sutural growth and may be responsible for the creation of malocclusions, the normal growth pattern should be better understood.

In the present study, apart from the ectocranial nasopremaxillary widths, all internasal and nasopremaxillary suture dimensions showed a significant decrease from 4 to 16 weeks of age. At 16 weeks of age, during adolescence, endocranial suture widths practically reached their final dimensions in both sutures. However, some significant alterations continue to occur in the ectocranial suture areas until 38 weeks of age. After 16 weeks of age, the changes in width differ between the internasal and nasopremaxillary ectocranial sutures. The nasopremaxillary ectocranial suture width tends to decrease from 16 to 38 weeks of age with the most significant decrease being observed after 26 weeks of age. However, the internasal ectocranial suture width showed a significant increase between 16 and 26 weeks of age, and then significantly decreased. The changes in the internasal suture width from 16 to 26 weeks of age in rats is in accordance with the changes in the interfrontal suture width in growing pigs (Sun et al., 2004), in which the endocranial width decreases, however the ectocranial width of the interfrontal suture tends to increase with age.

Sun et al. (2004), using a pig model, found that the endocranial suture widths were narrower than the ectocranial sides in the interfrontal and interparietal sutures. In the present study, apart from 4 weeks of age, the endocranial suture widths were narrower than the ectocranial sides in the internasal and nasopremaxillary sutures. The reason for the endocranial side being narrower might be because it is less influenced by masticatory strain, and suture widths reflect strain levels (Sun et al., 2004). According to the results of this study, the degree of suture closure differs between the ectocranial and endocranial surfaces, in accordance with the suggestions of Savoldi et al. (2019). In the present study it was shown that the endocranial sutures reach their final width at 16 weeks of age, while ectocranial width continues to change to some degree until 38 weeks of age.

The transversal dimensions of anterior viscerocranium were found to be strongly and negatively correlated with the endocranial and mean suture widths of the internasal suture, and the nasopremaxillary suture widths in both the endocranial and ectocranial region. Therefore, it can be hypothesized that bone apposition in the endocranial parts of the internasal suture is reflected more on the transversal dimensions of the anterior viscerocranium. Regarding the nasopremaxillary suture, although there was a strong and negative correlation in all regions, the strength of the correlation of the nasopremaxillary ectocranial suture width was less than the other regions according to the (r) values. Future studies might be conducted to understand the factors that affect endocranial and ectocranial suture widths, and their effect on craniofacial dimensions.

In the present study, fusion was seen in the ectocranial region of the internasal sutures in two 16-week-old rats and one 38-week-old rat. Engstrom et al. (1986) observed obliterative osteogenesis in the internasal sutures of 10 out of their 14 8-week-old soft-diet fed rats. The fact that they reported fusion in an earlier period and in more animals might imply that the reason of this obliteration may be the reduced masticatory function. However, fusion is also seen in normally-fed rats in the present study, but less frequently. This coincides with the knowledge that the internasal sutures of rats may stay patent throughout life, and this calls for future studies to better identify the reason for and the frequency of this obliteration.

The most significant change in the height of the internasal and nasopremaxillary sutures was observed as an increase from 4 to 16 weeks of age. Apart from this, a significant increase was also observed in internasal suture height during young adulthood. When combined with the changes in suture widths, these results might lead us to suggest that although the craniofacial dimensions reach their final dimensions following adolescence, some morphological changes continue to take place in some craniofacial sutures, even into young adulthood. Future studies might be conducted to understand if these sutures are still responsive to changing functional demands in the adult period, as shown in young rats (Katsaros et al., 1994; Katsaros et al., 2006).

Limitations of the present study are mainly related to its design. Since this study has a cross-sectional design, an exact time course of longitudinal dimensional changes of sutures during growth of a rat is not directly assessable. μCT imaging has the advantage of taking images without scarifying the animal, which allows us to repeatedly monitor the progress of suture development in a single animal, and thus further studies can be conducted with μCT imaging of the sutures by using growing rats with repeated imaging at different time periods. This can overcome the issue of between-animal differences that can be resulted from using different rats in a cross-sectional design. Another limitation of this study is that it was sex-specific, as all included rats were males. This fact improves the homogeneity of the sample, but at the same time might limit the generalisability of the results, as suture width changes may potentially be different in females.

## Conclusion

In male Wistar rats, although the internasal and nasopremaxillary endocranial suture widths nearly reach their final widths during adolescence, changes in the ectocranial and mean suture widths continue until early adulthood. The ectocranial suture width decreases with time in the nasopremaxillary suture. An increase in width however is observed in the internasal ectocranial suture width from 16 to 26 weeks of age, which can be related to an increased sutural growth rate in the ectocranial regions during adolescence. Regarding suture heights, although the most important changes take place between 4 and 16 weeks of age, a notable increase can also be observed in young adulthood, especially in the internasal suture. In the present study, the changes of internasal and nasopremaxillary suture dimensions were presented from childhood to early adulthood. The results of this study can be used as a reference to understand the differences in suture growth related to changes in functional demands.

## Data Availability

The raw data supporting the conclusion of this article will be made available by the authors, without undue reservation.
